# Can a Multi-Component Intervention Improve Pediatric Service Delivery in Guangzhou?

**DOI:** 10.3389/fpubh.2021.760124

**Published:** 2021-10-04

**Authors:** Fang Hu, Shuaijun Guo, Jianjun Lu, Ziang Li, Yanyan Song, Rafael Pérez-Escamilla, Suifang Lin, Yifei Hu

**Affiliations:** ^1^Department of Child Health, Guangzhou Women and Children's Medical Center, Guangzhou Medical University, Guangzhou, China; ^2^Centre for Community Child Health, Murdoch Children's Research Institute, Royal Children's Hospital, Melbourne, VIC, Australia; ^3^Department of Pediatrics, University of Melbourne, Melbourne, VIC, Australia; ^4^Department of Medical Affairs, The First Affiliated Hospital of Sun Yat-sen University, Guangzhou, China; ^5^Department of Child and Adolescent Health and Maternal Care, School of Public Health, Capital Medical University, Beijing, China; ^6^Office of Public Health Practice, Yale School of Public Health, Yale University, New Haven, CT, United States

**Keywords:** pediatric care, resource allocation, quality of health care, service efficiency, child mortality, intervention

## Abstract

**Background:** Accessible, equitable, and efficient pediatric service is critical to achieve optimal child health. This study aimed to evaluate the effectiveness of a multi-component intervention on the pediatric health system over two different periods in Guangzhou.

**Methods:** Based on the World Health Organization (WHO) “six building blocks” model and Donabedian's “Structure-Process-Outcomes” framework, an intervention package was developed to increase financial and human resouce investment to strengthen basic health care and strive for a better quality of pediatric care. This multi-component intervention package was conducted in Guangzhou to improve the pediatric service delivery during two stages (2011–2014 and 2016–2019). The main outcome indicators were the changes in the allocation of pediatricians and pediatric beds, pediatric service efficiency, and the impact of pediatricians on child mortality.

**Results:** We found that pediatricians per 1,000 children (PPTC) and pediatric beds per 1,000 children (PBPTC) increased from 1.07 and 2.37 in 2010 to 1.37 and 2.39 in 2014, then to 1.47 and 2.93 in 2019, respectively. Infant mortality rate (IMR) and under-5 mortality rate (U5MR) dropped from 5.46‰ and 4.04‰ in 2010 to 4.35‰ and 3.30‰ in 2014 then to 3.26‰ and 2.37‰ in 2019. The Gini coefficients of PPTC and PBPTC decreased from 0.48 and 0.38 in 2010, to 0.35 and 0.28 in 2014, then to 0.35 and 0.22 in 2019, respectively, representing the improvement of pediatric resources distribution according to service population. However, equalities in the spatial distribution were not improved much. The average efficiency of pediatric service fluctuated from 2010 to 2019. A unit increase in PPTC was associated with an 11% reduction in IMR and a 16% reduction in U5MR.

**Conclusions:** Findings suggest this multi-component intervention strategy is effective, particularly on the reduction of child mortality. In future, more rigorous and multi-faceted indicators should be integrated in a comprehensive evaluation of the intervention.

## Background

Child health is the foundation for the future development of child, family, community, and country (1). With the rapid improvement in social and economic conditions and the changes of disease spectrums among children, the focus of pediatric health care has shifted from a goal of “survival” to “thriving and prosperous”, resulting in growing demands for high-quality health care (2). Despite the significant reduction in child mortality globally, the increasing prevalence of premature, congenital abnormalities, injury, mental illness, and chronic conditions among children pose an unprecedented challenge to the present pediatric health care systems across countries (3), particularly in the context of coronavirus (COVID-19) pandemic. Strengthening pediatric service delivery is critical to achieve the health-related Millennium Development Goals, including the reduction of maternal mortality, child mortality, and disease burden resulting from HIV/AIDS, tuberculosis, and malaria (4).

Accessible, equitable, and efficient pediatric service is critical to achieve optimal child health (5). However, the shortage of pediatricians and inequalities in the distribution of pediatric resources exist in all countries, including developed countries. For example, there is a shortage of medical subspecialists in the field of pediatrics in the United States (6, 7). Similarly, almost one-half of the European countries have been reported of the decreasing numbers of in-service and pre-service pediatricians (8). In Asian countries like Japan, the pediatric health care system is also facing challenges of both shortage of pediatricians (9) and uneven geographic distribution (10). These common challenges across countries call for joint efforts from governments and health care organizations, such as reforming medical education and strengthening training to increase the pediatric workforce (7, 11–17).

China is a developing country undergoing rapid industrialization and social development with 253 million children under 14 years of age, accounting for 18.0% of the total population according to its most recent National Population Census (18). With the implementation of the universal three-child policy in 2021 (19), the number of births is expected to increase dramatically in future. Currently, the shortage of pediatricians and pediatric beds and uneven distributions of pediatric resources between rural and urban areas pose major challenges to meet the population's needs, flagging a significant health and social issue in China (3, 20, 21). Results from national surveys showed that the number of pediatricians per 1,000 children (PPTC) up to age 14 was 0.40 in China, which was only half of that in Japan (PPTC = 0.93) and a quarter of that in USA (PPTC = 1.90) (3). Furthermore, the distribution of pediatricians was extremely uneven, with rural areas having a higher shortage (PPTC = 0.39) (3). Because the pediatric department does not have capacity of profit making, the pediatric department of general hospitals has been neglected over the past decade, and many general hospitals have even closed pediatric inpatient service (22).

To help inform evidence-based decision-making about better pediatric service delivery, there have been a number of studies exploring the underlying causes that lead to the shortage and inequalities in pediatric resources in China. This includes barriers at multiple levels including patients [e.g., poor medical adherence, high demand for health care services (21)], health providers (e.g., low cultural competence, heavy workload) (3, 20–24), and health care systems [e.g., lack of workforce resources, institutional bias, set physicians' salary based on their specialties/subspecialties (20, 21)]. In order to address these barriers and achieve an accessible and equitable pediatric health care, the Chinese government issued a policy entitled “Strengthening the reform and development of pediatric health care service delivery” in 2016 (25). Of particular note, this policy document sets a goal of “increasing the number of pediatricians and pediatric beds per 1,000 children (PBPTC) up to age 14 to 0.69 and 2.2 by 2020, respectively” (25).

**Figure 1:**
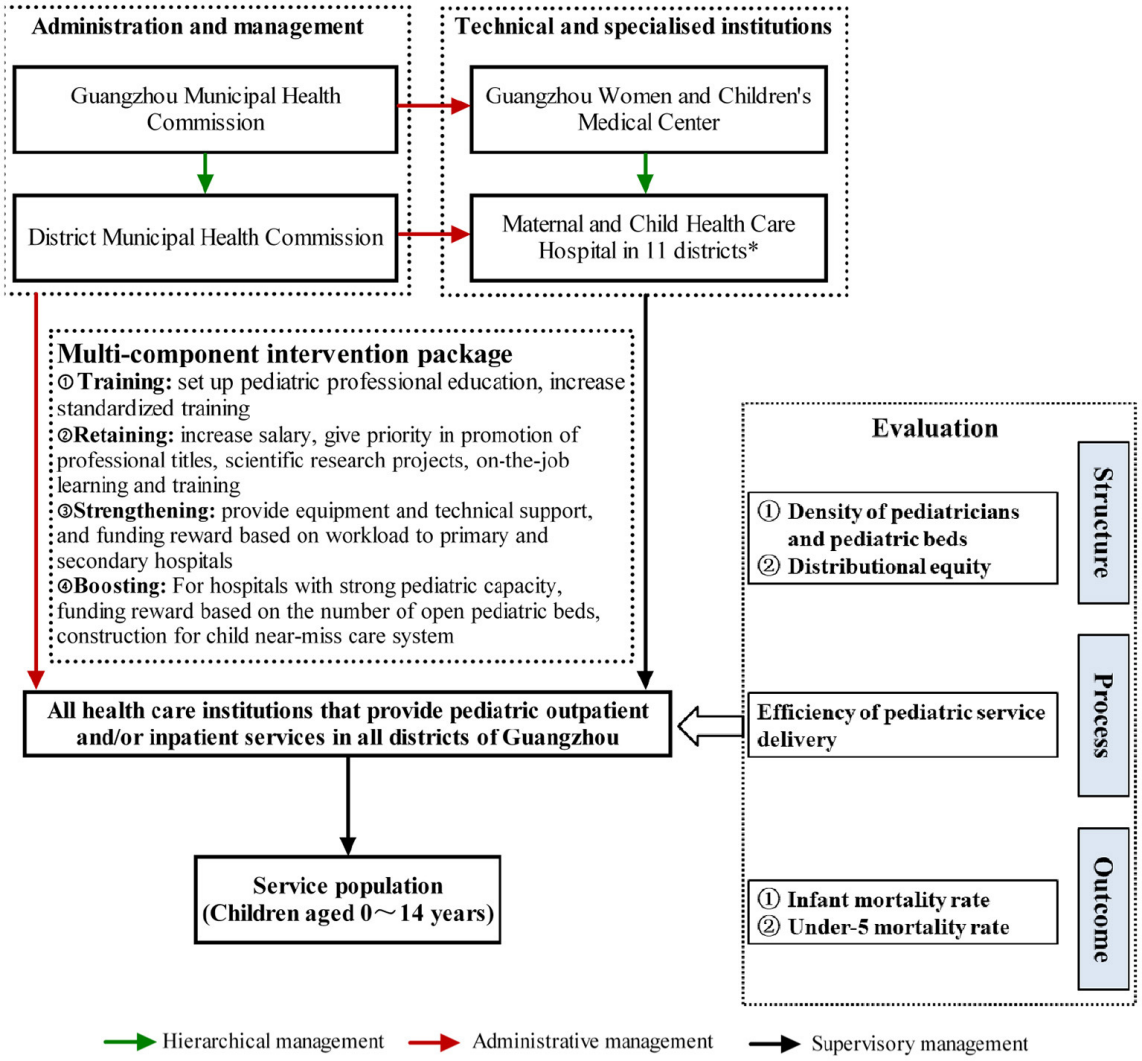
Triple management network for maternal and child health care and strategies and evaluation framework of the two-stage intervention.

As one of the most prosperous cities in China, Guangzhou has 2.59 million resident children up to age 14, accouting for 13.87% of the resident population in the city (26). The pediatric service delivery in Guangzhou is also facing challenges such as lack of pediatricians and pediatric beds, and their uneven geographical distribution. Guangzhou government has set an even more ambitious goal of having ratios of PPTC of 1.3 and of PBPTC of 2.9 by 2020. To achieve this goal, Guangzhou government launched a comprehensive health care program in 2011, of which a multi-component intervention package was implemented in two stages (January 2011 to October 2014 and January 2016 to December 2019). This intervention package was derived from the World Health Organization (WHO) “six building blocks” model (4) and Donabedian “Structure-Process-Outcomes” framework (27) (Figure 1, see further details in Methods section). Compared to a single-component intervention strategy, the present strategy is preferred because it concurrently addresses improvements in both the quality and equity of pediatric service delivery. As demonstrated in previous empirical research, multi-component interventions showed better outcomes at both individual and system levels (28).

To the best of our knowledge, there is little evidence regarding the implementation of a multi-component intervention on pediatric service delivery in China and whether it is effective in practice. In the present study, we aim to examine the effectiveness of a multi-component intervention that seeks to improve the quality and equity of pediatric service delivery in Guangzhou. Specifically, there are three objectives to be accomplished, including: (1) assessing the density of pediatricians and pediatric beds and the equalities in the distribution of these resources over time; (2) evaluating the efficiency of pediatric service delivery over time; and (3) investigating the relationship between pediatricians density and child mortality over time.

## Methods

### Study Design and Participants

We used a two-stage intervention study design to evaluate the effectiveness of a multi-component intervention package implemented in Guangzhou. All health care institutions that provide pediatric outpatient and/or inpatient services in all districts (*n* = 11) of Guangzhou participated in this study.

In China, a pediatrician is defined as a physician certified by the National Health Commission of the People' s Republic of China and licensed as specializing in medical care for children. Open pediatric beds refer to beds that provide inpatient services for children. The resident children analyzed in this study refer to children aged 0–14 years who have lived in Guangzhou for 6 months or more, which is consistent with the resident children definition from the Chinese Statistics Bureau and the Chinese traditional pediatrician practice (3).

### Intervention Model and Its Components

A multi-component intervention package was developed (Figure 1) based on the WHO “six building blocks” model (4) and Donabedian's “Structure-Process-Outcomes” framework (27). While the WHO “six building blocks” model does not address the underlying social determinants of health, it is helpful to put boundaries between each component in the complex health care system and inform measurement strategies for monitoring and evaluating health system strengthening efforts. Donabedian's “Structure-Process-Outcomes” framework was also chosen because it is generally the touchstone for evaluating the quality of health care (27). In the present study, “structure” is defined as the allocation of pediatricians and pediatric beds in medical institutions, “process” refers to the operational efficiency of medical institutions, and “outcomes” denote the effects of pediatric service delivery on the child mortality.

Specifically, we used the WHO “six building blocks” model (4) as our guideline to determine the intervention components, which included “Training”, “Retaining”, “Strengthening”, and “Boosting”. “Training” measures included setting up full-time major in clinical pediatric medicine and establishing financial incentive mechanisms to increase standardized training of pediatricians. “Retaining” measures included increasing income, assigning priority for support for scientific research projects and professional promotion, and providing various training opportunities for the productive pediatricians to retain them. In terms of “strengthening”, we provided facilities/equipment and technical support to health facilities at grassroot level, and formulated a policy of performance bonus system based on workload. Regarding “boosting”, we focused on further enhancing capacity of medical institutions with strong pediatric capacities, formulated an institutional rewarding system based on the number of open pediatric beds, and constructed municipal-level and district-level child near-miss care centers.

This multi-component intervention package was implemented in two phases (January 2011–October 2014, and January 2016–December 2019). In the first stage, we mainly carried out training for on-the-job personnel in “retaining”, equipment and technical support in “strengthening” for first-level hospitals, and establishing municipal-level and district-level child near-miss care centers in “boosting”. In the second stage, we continued to improve the first stage of initiatives and focused on second-class hospitals. More importantly, Guangzhou Municipal Health Commission and Guangzhou Municipal Finance Bureau jointly promulgated a series of policies to increase the number of pediatricians trained, improve the income of on-the-job pediatricians, and mobilize medical institutions to pay attention to pediatrics development. The specific information about the pediatric intervention package is presented in Table 1.

**Table 1:** Strategies of the two-stage intervention for improving the capacity of pediatric service delivery in Guangzhou, China, 2011–2019.

**Stage 1 (January 1, 2011–October 31, 2014)**	**Stage 2 (January 1, 2016–December 31, 2019)**
**A. Training**	
**Goal: increase the number of newly trained young pediatricians**	
Not applicable	**1**) **Guangzhou Medical University set up full-time majors in clinical pediatrics medicine** Goal: Training 200 pediatric graduates • Strategy: Reduce tuition and fees **2**) **Expand the scale of standardized residency training for pediatricians** • Goal: Training 400 pediatricians • Strategy: Give annual living allowance of 30,000 RMB
**B. Retaining**	
**Goal: Retain on-the-job pediatricians**	
**1**) **Carry out various professional skill training to improve the capability of pediatricians** • **Off-the-job training for 3 months in designated tertiary hospitals with completion exams** Target subjects: 100 neonatologists from primary hospitals in 10 districts excluding Yuexiu were trained. • **Pediatric advanced life support training** Target subjects: All pediatricians (over 800) from primary and secondary hospitals were trained. Training skills: 2010 international consensus on cardiopulmonary resuscitation and emergency cardiovascular care scientific skills with treatment recommendations Training method: theory lectures, demonstration, drills, and simulation exercises • **Neonatal resuscitation training** Target subjects: Backbones of neonatologists from all hospitals in 11 districts Training skills: Neonatal Resuscitation Guidelines (Revised in 2011) Training method: operation drills using models Training frequency: once a year • **Regular professional training** Target subjects: neonatologists and pediatricians from all hospitals in 11 districts Target skills: up-to-date pediatric clinical knowledge and skills	**1**) **Carry out various professional skill training to improve the capability of pediatricians** • **Off-the-job training for 2 months in designated tertiary hospitals with completion exams** Target subjects: 100 pediatricians from secondary hospitals in all 11 districts were trained • **Pediatric advanced life support training** Target subjects: Training all pediatricians from all healthcare institutions. Training skills: 2015 international consensus on cardiopulmonary resuscitation and emergency cardiovascular care scientific skills with treatment recommendations Training method: theory lectures, demonstration, drills, and simulation exercises • **Neonatal resuscitation training** Target subjects: All neonatologists from all hospitals in 11 districts. Target skills: Neonatal Resuscitation Guidelines (Revised in 2016) Training method and frequency: same as Stage 1 • **Regular professional training** Same as Stage 1 • **Standardization construction of municipal and district-level maternal and child health skill Training bases** Goal: Improving the hardware equipment configuration of the training bases Strategy: Set up municipal and district-level teaching team **2**) **Improve the salary of pediatricians** • 30,000 RMB for pediatricians newly recruited in Guangzhou municipal medical institutions • The salary of pediatricians must exceed 20% of the average at the same level of staff in the same hospital. • Reasonably adjust the prices of pediatric medical service to respect the labor value of pediatricians. **3**) **Promote the professional development of pediatricians** • Guangzhou government gives priority to support pediatrics in discipline construction, talent training, scientific research projects, funding arrangement, and so on.
**C. Strengthening**	
**Goal: strengthen the capacity of pediatrics in primary and secondary medical institutions**	
**1**) **Construction of pediatrics specialty of basic midwifery institutions** Goal: 24 primary hospitals supported, covering seven rural districts Strategy: Configure basic pediatric equipment, such as neonatal monitors, baby ventilator, among others **2**) **Provided with basic equipment for emergency treatment of newborns** • Goal: 68 primary hospitals supported, covering nine districts • Strategy: The government provided free basic equipment for emergency treatment of newborns	**1**) **Construction of pediatrics specialty of secondary hospitals** Goal: 30 secondary hospitals supported, covering 11 districts Strategy: The government provided free basic equipment for emergency treatment of newborns **2**) **Establish a special subsidy mechanism for pediatrics** Secondary and primary hospitals receive government funding subsidies based on the increase of pediatric outpatient and inpatient visits every year.
**3**) **Expert on-site instructions** Goal: Experts were assigned to 82 primary and secondary health care institutions in 10 districts excluding Yuexiu, each expert working on site for 100 days. Strategy: Pediatricians with senior titles in 22 tertiary hospitals helped primary and secondary hospitals through teaching, thematic training, complicated case discussion, surgical demonstration, and so on	
**D. Boosting**	
**Goal: further enhancing capacity of medical institutions with strong pediatric potential, mainly tertiary hospitals, and some secondary maternal and child health care hospitals with strong pediatric service capacity**	
**1**) **Capacity building for the child near-miss care system** • Establish four new municipal-level and eight new district-level child near-miss care centers on the demand • Establish “green channel” (expedited service access and appointment) for referral of critically ill children	**1**) **Capacity building for the near-miss child care system** • Further strengthen the capacity of municipal-district level near-miss child care centers **2**) **Establish a special subsidy mechanism for pediatrics** • Guangzhou government subsidizes tertiary hospitals, according to the annual increase in the number of open pediatric beds

### Study Indicators

#### Structural Indicators

We analyze the structure of pediatric service delivery from two perspectives (i.e., the density and distributional equity of pediatricians and pediatric beds). The density of pediatricians and pediatric beds refers to the numbers of PPTC, PBPTC, pediatricians per 100 km^2^ area (PPHA), and pediatric beds per 100 km^2^ area (PBPHA). The distributional equity in the allocation of pediatricians and pediatric beds is analyzed from the perspectives of service population and geographic area.

#### Process Indicators

In terms of process indicators, we analyze the efficiency of pediatric service delivery that is the core element of the health system.

#### Outcome Indicators

This study analyzes the impact of pediatricians density on child mortality that includes infant mortality rate (IMR) and under-5 mortality rate (U5MR).

### Data Collection

We collected data from multiple standardized government healthcare statistics data sources recommended by WHO (4): Guangzhou Statistics Yearbook, Guangzhou Annual Report of Medical Institutions and Health Statistics, and Maternal and Child Mortality Surveillance system. The number of resident children under 14 years of age was obtained from the Guangzhou Statistics Yearbook. Numbers of pediatricians, pediatric beds, and pediatric outpatient and inpatient visits were obtained from the Guangzhou Annual Report of Medical Institutions and Health Statistics by the Guangzhou Municipal Health Commission. IMR and U5MR were obtained from the Maternal and Child Mortality Surveillance system. The data in 2010 were used as the baseline data before the intervention, and the data in 2014 and 2019 presented the results post stage-one and stage-two intervention, respectively.

Each medical institution reported numbers of pediatricians and pediatric beds in the previous year, and numbers of pediatric outpatient and inpatient visits in the previous quarter to the Maternal and Child Health Hospital of the jurisdiction at the beginning of each year or quarter. After verified and reviewed by the Maternal and Child Health Hospital of the jurisdiction and the Guangzhou Women and Children's Medical Center, all data were reported to the Guangzhou Municipal Health Commission. Death cause of each child case under the age of 5 is compulsory to be analyzed in detail by the hospital, district, and municipal review committees, and then reviewed and reported to the Guangzhou Municipal Health Commission.

### Statistical Analysis

The density of pediatricians and pediatric beds (i.e., PPTC, PPHA, PBPTC, and PBPHA) of Guangzhou maps was conducted using R software (V3.6.2, R Foundation for Statistical Computing, Vienna, Austria) ggplot2 package. Distributional equity was evaluated using the Gini coefficient (G), which was derived from the Lorenz curve (29). The Gini coefficient is a quantitative indicator ranging from 0 to 1. In general, G < 0.3 is considered to be equal, 0.3 ≤ G < 0.4 relatively unfair, and G > 0.4 to be unfair.

Pediatric service efficiency was assessed using the super-Slacks-based data envelopment analysis model (DEA), which presented a structure of multiple inputs and outputs (30). The numbers of pediatric healthcare facilities, pediatricians, and pediatric beds were selected as input indicators. The numbers of outpatient and inpatient visits were modeled as output indicators. Efficiency score ≥1.0 means efficient, <1.0 means inefficient, and higher efficiency score, more efficient is (31). The efficiency analysis was conducted with R software, deaR package.

Fixed-effects Poisson regression model was used to predict the association between PPTC and U5MR or IMR, respectively, and the effects were estimated with the natural log of the live births and an offset parameter was generated using SAS software (V9.4, SAS Institute, Cary, NC, USA). We considered the districts of Guangzhou and year of data collection as fixed effects to control for time-invariant district-specific factors. In addition, considering that a large number of migrant children or children from other cities/provinces come to Guangzhou for medical service each year, we performed a sensitivity analysis to test the robustness of the results. We specifically modeled the population compositional changes of resident children to migrant children served by pediatricians. A value of *P* < 0.05 (two-tailed tests) was considered statistically significant.

## Results

### The Density and Distributional Equity of Pediatricians and Pediatric Beds in Guangzhou

In 2010, there were 1,549 pediatricians in Guangzhou, and the numbers of PPTC and PPHA were 1.07 and 20.84, respectively. In 2014 and 2019, the number of pediatricians in Guangzhou increased to 2,327 and 2,823, representing growth rates of 50.23 and 82.25% (Supplementary Table 1). The numbers of PPTC in 2014 and 2019 were 1.37 and 1.47 and for PPHA were 31.30 and 37.97, respectively.

In 2010, there were 3,448 pediatric beds in Guangzhou, and the numbers of PBPTC and PBPHA were 2.37 and 46.38, respectively. In 2014 and 2019, the number of pediatric beds in Guangzhou increased to 4,081 and 5,634, representing growth rates of 18.36 and 63.40% compared with 2010 (Supplementary Table 2). The corresponding numbers of PBPTC in 2014 and 2019 were 2.39 and 2.93, while PBPHA were 54.89 and 75.78, respectively.

**Figure 2:**
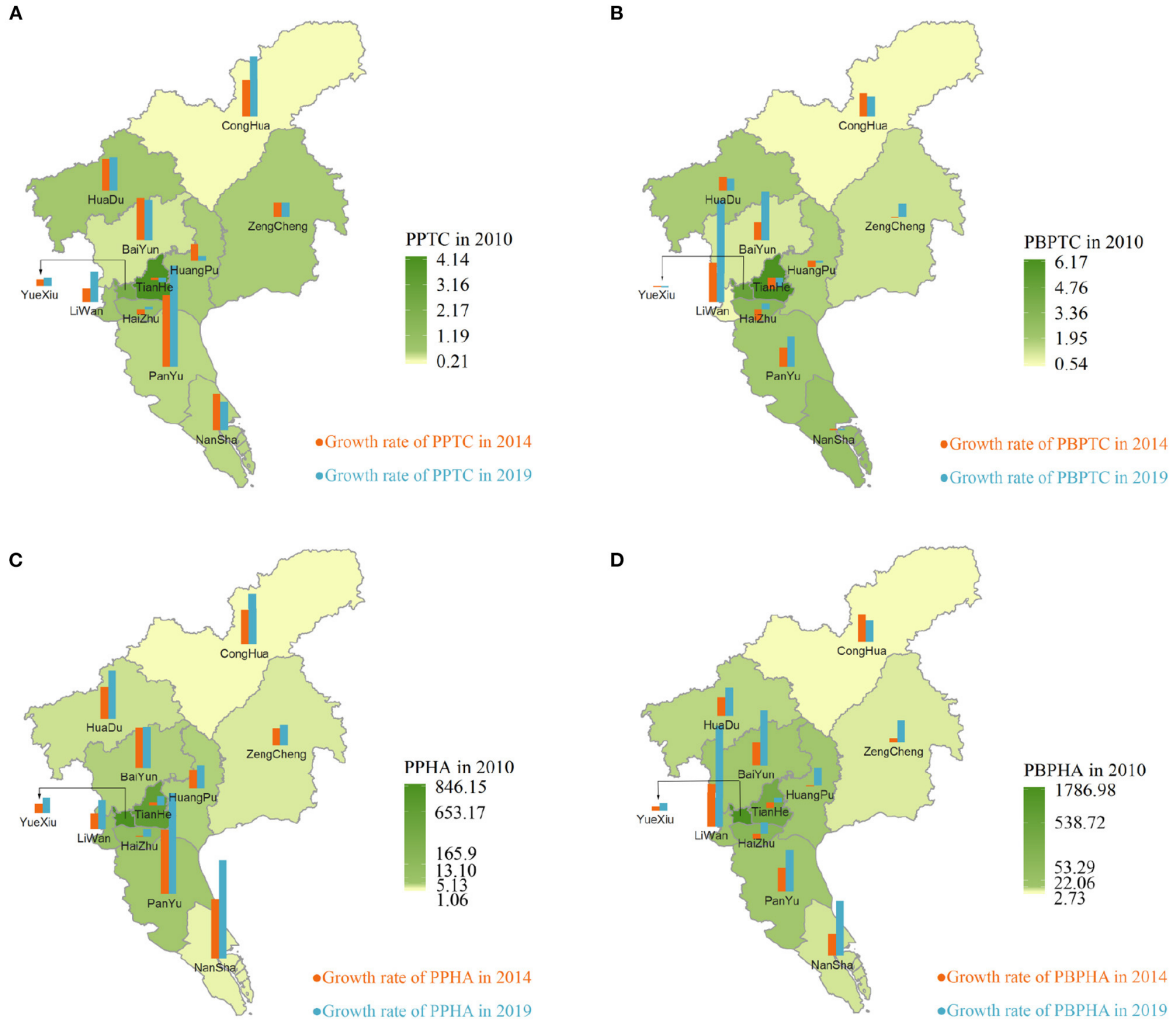
Spatial clusters and changes of pediatricians and pediatric beds per share of geographic area and children aged 0–14 years old in 11 districts in Guangzhou. **(A)** Pediatricians distribution per share of children aged 0–14 years old, **(B)** pediatric beds distribution per share of children aged 0–14 years old, **(C)** pediatricians distribution per share of geographic area, and **(D)** pediatric beds distribution per share of geographic area. The hot spots indicate the spatial clusters with many pediatric beds and pediatricians. The cold spots indicate the spatial clusters with few pediatric beds and pediatricians.

Substantial allocation inequities were observed in the distribution of pediatricians and pediatric beds in 11 districts (Figure 2). From 2010 to 2019, the numbers of PPTC, PPHA, PBPTC, and PBPHA were highest in the central areas (including Yuexiu, Tianhe, Liwan, and Haizhu). Interestingly, the numbers of PPTC and PBPTC in the semi-urban areas (including Baiyun and Panyu) were higher than those in the rural areas (including Huadu, Nansha, Zengcheng, and Conghua) in 2010. By contrast, the numbers of PPHA and PBPHA were smaller in rural areas, and this pattern remained unchanged in 2014 and 2019.

**Figure 3:**
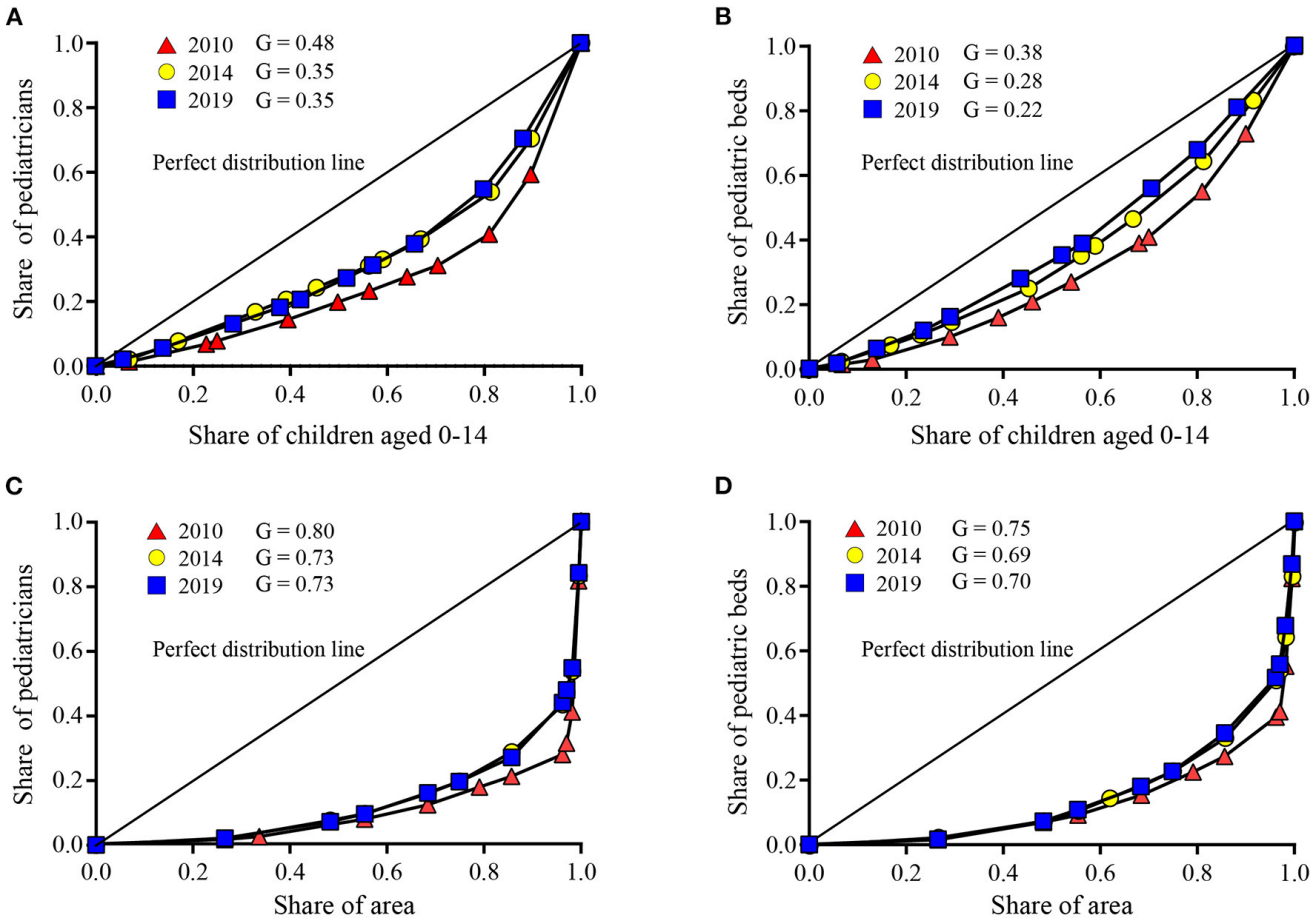
Lorenz curves for pediatricians and pediatric beds distribution per share of geographic area and children aged 0–14 years old. **(A)** Pediatricians distribution per share of children aged 0–14 years old, **(B)** pediatric beds distribution per share of children aged 0–14 years old, **(C)** pediatricians distribution per share of geographic area, and **(D)** pediatric beds distribution per share of geographic area illustrate the gap between the real and ideal distributions for the 3 years.

From 2010 to 2019, the Lorenz curves depicting pediatricians and pediatric beds per share of children were close to the equity line (Figures 3A,B). The Gini coefficients for pediatricians and pediatric beds per share of children in 2010, 2014, and 2019 were 0.48, 0.35, and 0.35; and 0.38, 0.28, and 0.22, respectively, indicating that equity in the distribution of pediatricians and pediatric beds across different districts per children improved over time.

Unlike the curves for distribution by children served, the Lorenz curves showing pediatricians and pediatric beds per geographical area were far from the ideal equity line, as the Gini coefficients in 2010, 2014, and 2019 were 0.80, 0.73, and 0.73; and 0.75, 0.69, and 0.70, respectively (Figures 3C,D). This finding indicates first, pediatricians and pediatric beds per geographical area were unequally distributed across different districts, and second, this inequity did not narrow between 2014 and 2019.

### The Efficiency of Pediatric Service Delivery

As shown in Table 2, the average efficiency of pediatric service delivery fluctuated from 2010 to 2019. In 2010, the average service efficiency estimate was 1.12; then it decreased to 1.09 in 2014; and subsequently increased to 1.13 in 2019. Across districts, the efficiency estimate in Liwan, Tianhe, Panyu, Huadu, and Conghua decreased from 2010 to 2019, the efficiency estimate in Haizhu, Baiyun, Huangpu, and Nansha remained unchanged, and only that in Yuexiu increased. Zengcheng trajectory fluctuated between 1.19 in 2020, 1.31 in 2014, and 1.06 in 2019.

**Table 2:** Changes of pediatric service efficiency of each district in Guangzhou, China, 2010–2019.

**District**	**2010**	**2014**	**2019**	**Average**
Liwan	1.70	1.00	1.00	1.24
Yuexiu	1.16	1.65	2.32	1.71
Haizhu	1.00	1.00	1.00	1.00
Tianhe	1.04	1.00	1.00	1.01
Baiyun	1.00	1.00	1.00	1.00
Huangpu	1.00	1.00	1.00	1.00
Panyu	1.03	1.00	1.00	1.01
Huadu	1.03	1.00	1.00	1.01
Nansha	1.00	1.00	1.00	1.00
Zengcheng	1.19	1.31	1.06	1.19
Conghua	1.17	1.06	1.00	1.07
Average	1.12	1.09	1.13	1.11

### The Impact of Pediatrician Density on Child Mortality Rate

The U5MR in Guangzhou was 5.46‰ in 2010, 4.35‰ in 2014, and 3.26‰ in 2019, representing a 40.29% decrease in the past decade. The IMR was 4.04‰ in 2010 and 2.37‰ in 2019, representing a decrease of 41.34%. Table 3 shows that the PPTC is inversely associated with U5MR and IMR. On average, a unit increase in PPTC is associated with a 16% reduction in U5MR, a 11% reduction in IMR.

**Table 3:** Effects of the pediatrician density on under-5 mortality and infant mortality rate respectively using a Multivariate Poisson Regression Model in Guangzhou, 2010–2019 (*n* = 33).

**Dependent variable**	**Independent variable**	**Estimate**	**SE**	**IRR (95%CI)**	***p*-value**
Under-5 mortality rate	Pediatrician density	−0.170	0.03	0.84 (0.79–0.90)	<0.001
	Year (ref = 2010)				
	2014	−0.169	0.061	/	0.006
	2019	−0.445	0.078	/	<0.001
Infant mortality rate	Pediatrician density	−0.118	0.040	0.89 (0.82–0.96)	0.003
	Year (ref = 2010)				
	2014	−0.163	0.087	/	0.062
	2019	−0.487	0.090	/	<0.001

*Both models were adjusted with year respectively. SE, standard error; IRR, incidence rate ratio*.

We conducted sensitivity analyses considering the large migrant population in Guangzhou, and the fact that pediatricians also need to provide standard medical services for migrant children. The sensitivity analyses presented in Supplementary Table 3 showed that the relationship, especially the directionality of the association between PPTC and U5MR, and PPTC and IMR remained unchanged.

## Discussion

This study was the first, to our knowledge, to evaluate the effect of a two-stage multi-component intervention on improving the coverage and quality of pediatric service delivery in Guangzhou and in China. Our intervention showed promising outcomes for the pediatric service delivery from 2011 to 2014 and from 2016 to 2019. Specifically, we found that: (1) the numbers of pediatricians and pediatric beds per share of children and geographical area increased significantly coinciding with the implementation of the two-stage intervention; (2) the equity in the distribution of pediatricians and pediatric beds per served population was equal and improved over these years, while the distribution per geographical area remained inequitable; (3) the average efficiency of pediatric service fluctuated from 2010 to 2019, and the difference over time was small in most districts; (4) U5MR and IMR in Guangzhou decreased significantly and was inversely associated with the PPTC.

During the two-stage intervention's implemention, there has been a significant increase in the numbers of pediatricians and pediatric beds over the last decade in Guangzhou. Inconsistent from previous findings (22), we found workforce resources (pediatricians) increased relatively faster than the infrastructure (pediatric beds). The PPTC in Guangzhou in 2019 (1.47) reaches the 2020 target goal in Guangzhou, but it is still smaller than that of the United States (1.9) (3). Our study showed that the incremental number of pediatricians was mainly located in tertiary hospitals in the Panyu, Nansha, Baiyun, Conghua, or Huadu district, which have huge economic development potential. Many factors can affect the geographic registration of pediatricians in different hospitals, including career development prospects, training opportunities, living and working convenience, financial incentives (32, 33).

Optimizing the distribution of pediatric service delivery is a major challenge, because the uneven distribution is generally driven by socioeconomic inequities (3, 10). Our study found that although the numbers of pediatricians and pediatric beds per share of children improved, there were still striking differences across geographical areas, which is similar with the situation in USA (34) and Japan (10, 35). This is a long-lasting issue that has been now of concern to policy makers, awaiting for fundamental and sustainable solutions (10, 36). When the density of pediatricians is stable, the larger the geographic area served by each pediatrician, the less effective the service provided (37). In future, policy makers should consider the impact of geographical factors on the accessibility to health service. Specifically, it is critical to focus on investing in capacity building through pediatric workforce development in the suburban and rural areas. Economic incentives such as more attractive salaries, performance-based bonuses, and on-the-job training have shown successful implementations in rural areas (29).

Efficiency reflects the relationship between outputs and inputs of the health system (31), and is a strong prerequisite for attaining equity in access to services (30). Though Guangzhou has increased the health investment in rural areas drastically, we did not find corresponding efficiency increase over time. This suggests that continued efforts are needed to balance the “improvement of outputs” and “increasing inputs” for policy makers. For example, during the implementation process of our intervention, new equipments were not used properly or even never used in rural areas because pediatricians rarely treat serious patients (they don't want to risk any failure and rather tranfer the complicated cases to the upper level hospitals). However, the measure of efficiency for the pediatric service system is complex. We did not include input and output indicators such as the pediatric assets of each facility and inpatient expenditures. Future research is needed to integrate more robust and comprehensive indicators to investigate the efficiency of pediatric service delivery.

Child mortality is a sentinel surveillance indicator, reflecting the well-being of mothers, families and of countries development (38). The U5MR in Guangzhou decreased from 5.46‰ in 2010 to 3.26‰ in 2019, which compares favorably with countries with advanced economies (39) [Changes in the U5MR, source from UN Interagency Group for Child Mortality Estimation in 2019 http://www.childmortality.org (21)]. The accessibility and quality improvement of pediatric service delivery are key factors influencing the child mortality (5, 22). Our findings show that each extra unit increase in the number of PPTC is associated with a U5MR decline of 16%. Consistent with this finding, another study in Japan also showed that a unit increase in pediatrician density was associated with a 7% reduction in U5MR (16).

Our study has several strengths. First, it is the first study to evaluate the impact of multi-component interventions on the pediatric service delivery in China. These interventions are derived from the WHO's “six building blocks” model and comprehensively improve pediatric service delivery from four aspects including “Training”, “Retaining”, “Strengthening”, and “Boosting”. Second, we comprehensively evaluated the improvement of the pediatric service delivery in terms of resource allocation, utilization efficiency and effect on child mortality based on widely used “structure-process-outcome” framework. Results from this two-stage intervention are consistent, indicating the robustness of our findings and the effectiveness of this multi-component intervention. Third, we applied visualization of results and comparatively new methods of statistical analysis. This study used the super-Slacks-based DEA to evaluate the efficiency of pediatric service, which avoids the shortcomings of traditional DEA.

However, there are a few limitations. First, we conducted a before-and-after intervention study to evaluate the effectiveness. Quasi-experimental studies or randomized controlled trials are needed in future to examine whether such multi-component interventions are effective or not. Second, the PPTC and PBPTC refer to the numbers of pediatricians and pediatric beds per 1,000 resident children, excluding migrant children and children who visit Guangzhou for medical service. Those children may compete with resident children in access to pediatric care (40). However, our sensitivity analysis found it unchanged after assuming different migrant to resident ratios. Third, the determinants of efficiency in the medical service system were complicated (31), and some factors were not measured or incorporated into the analysis such as pediatric assets and inpatient expenditure, which may affect the result. Fourth, this study was only carried out in one mega developed city in China, which may affect the generalisability of our findings.

## Conclusions

Our encouraging findings suggest that investment in pediatric service delivery can lead to improvements on child health outcomes. Specifically, the quantity and quality of pediatric service delivery in Guangzhou were improved in response to the two-stage intervention. However, the distribution of pediatricians and pediatric beds per geographical area did not improve. We recommends that the government should provide preferential policies for remote rural areas in future. In addition, given that Guangzhou has a high proportion of migrant children, the government should continue to improve the pediatric service capacity in Guangzhou. Lastly, considering the above limitations of this study, further research may consider using quantitative indicators as well as qualitative information to evaluate and monitor the development progress of health service delivery.

## Data Availability Statement

The raw data supporting the conclusions of this article will be made available by the authors, without undue reservation.

## Author Contributions

FH carried out the entire intervention process, performed the initial analyses, and drafted the initial manuscript. YH and SL conceptualized and designed the study, and critically reviewed the manuscript. SG and RP-E critically reviewed the manuscript. JL and YS coordinated and supervised data collection, and critically reviewed the manuscript. ZL performed the initial analyses and drafted the initial manuscript. All authors have provided input into and approved the final manuscript.

## Funding

This study was supported by the National Natural Science Foundation of China (82073574) and Guangzhou Institute of Pediatrics/Guangzhou Women and Children's Medical Center (YIP-2019-001). The funders had no role in the design of the study and will have no role in data collection, analysis, interpretation, or manuscript writing.

## Conflict of Interest

The authors declare that the research was conducted in the absence of any commercial or financial relationships that could be construed as a potential conflict of interest.

## Publisher's Note

All claims expressed in this article are solely those of the authors and do not necessarily represent those of their affiliated organizations, or those of the publisher, the editors and the reviewers. Any product that may be evaluated in this article, or claim that may be made by its manufacturer, is not guaranteed or endorsed by the publisher.
