# Supplementary material for: Can a Multi-Component Intervention Improve Pediatric Service Delivery in Guangzhou?
Source: Front Public Health. 2021 Oct 4;9:760124. doi: 10.3389/fpubh.2021.760124 (PMC8520973; doi:10.3389/fpubh.2021.760124)
Supplement: Supplementary file 1 [file Table_1.DOCX]

**Table 1 Changes of pediatricians in different levels of hospitals in 11 districts, Guangzhou**

| District | Year | Primary hospitals | |  | Secondary hospitals | |  | Tertiary hospitals | |  | All | |
| --- | --- | --- | --- | --- | --- | --- | --- | --- | --- | --- | --- | --- |
|  |  | Pediatri-cians | Growth rate (%) |  | Pediatricians | Growth rate (%) |  | Pediatr-icians | Growth rate (%) |  | Pediatr-icians | Growth rate (%) |
| Liwan | 2010 | 1 |  |  | 30 |  |  | 24 |  |  | 55 |  |
|  | 2014 | 2 | 100.00 |  | 48 | 60.00 |  | 36 | 50.00 |  | 86 | 56.36 |
|  | 2019 | 4 | 300.00 |  | 56 | 86.67 |  | 53 | 120.83 |  | 113 | 105.45 |
| Yuexiu | 2010 | 0 |  |  | 50 |  |  | 236 |  |  | 286 |  |
|  | 2014 | 0 |  |  | 71 | 42.00 |  | 312 | 32.20 |  | 383 | 33.92 |
|  | 2019 | 0 |  |  | 108 | 116.00 |  | 337 | 42.80 |  | 445 | 55.59 |
| Haizhu | 2010 | 2 |  |  | 29 |  |  | 119 |  |  | 150 |  |
|  | 2014 | 0 | -100.00 |  | 26 | -10.34 |  | 130 | 9.24 |  | 156 | 4.00 |
|  | 2019 | 10 | 400.00 |  | 34 | 17.24 |  | 146 | 22.69 |  | 190 | 26.67 |
| Tianhe | 2010 | 11 |  |  | 26 |  |  | 592 |  |  | 629 |  |
|  | 2014 | 11 | 0.00 |  | 50 | 92.31 |  | 628 | 6.08 |  | 689 | 9.54 |
|  | 2019 | 11 | 0.00 |  | 58 | 123.08 |  | 763 | 28.89 |  | 832 | 32.27 |
| Baiyun | 2010 | 9 |  |  | 46 |  |  | 31 |  |  | 86 |  |
|  | 2014 | 27 | 200.00 |  | 92 | 100.00 |  | 92 | 196.77 |  | 211 | 145.35 |
|  | 2019 | 25 | 177.78 |  | 65 | 41.30 |  | 123 | 296.77 |  | 213 | 147.67 |
| Huangpu | 2010 | 13 |  |  | 40 |  |  | 0 |  |  | 53 |  |
|  | 2014 | 12 | -7.69 |  | 36 | -10.00 |  | 41 |  |  | 89 | 67.92 |
|  | 2019 | 13 | 0.00 |  | 36 | -10.00 |  | 49 |  |  | 98 | 84.91 |
| Panyu | 2010 | 43 |  |  | 60 |  |  | 0 |  |  | 103 |  |
|  | 2014 | 45 | 4.65 |  | 19 | -68.33 |  | 277 |  |  | 341 | 231.07 |
|  | 2019 | 55 | 27.91 |  | 26 | -56.67 |  | 396 |  |  | 477 | 363.11 |
| Huadu | 2010 | 15 |  |  | 53 |  |  | 0 |  |  | 68 |  |
|  | 2014 | 38 | 153.33 |  | 68 | 28.30 |  | 39 |  |  | 145 | 113.24 |
|  | 2019 | 57 | 280.00 |  | 20 | -62.26 |  | 108 |  |  | 185 | 172.06 |
| Nansha | 2010 | 15 |  |  | 0 |  |  | 0 |  |  | 15 |  |
|  | 2014 | 18 | 20.00 |  | 19 |  |  | 10 |  |  | 47 | 213.33 |
|  | 2019 | 29 | 93.33 |  | 22 |  |  | 17 |  |  | 68 | 353.33 |
| Zengcheng | 2010 | 33 |  |  | 50 |  |  | 0 |  |  | 83 |  |
|  | 2014 | 38 | 15.15 |  | 75 | 50.00 |  | 20 |  |  | 133 | 60.24 |
|  | 2019 | 40 | 21.21 |  | 79 | 58.00 |  | 24 |  |  | 143 | 72.29 |
| Conghua | 2010 | 3 |  |  | 18 |  |  | 0 |  |  | 21 |  |
|  | 2014 | 7 | 133.33 |  | 40 | 122.22 |  | 0 |  |  | 47 | 123.81 |
|  | 2019 | 13 | 333.33 |  | 8 | -55.56 |  | 38 |  |  | 59 | 180.95 |
| **Total** | **2010** | **145** |  |  | **402** |  |  | **1002** |  | **0** | **1549** |  |
|  | **2014** | **198** | **36.55** |  | **544** | **35.32** |  | **1585** | **58.18** | **0** | **2327** | **50.23** |
|  | **2019** | **257** | **77.24** |  | **512** | **27.36** |  | **2054** | **104.99** | **0** | **2823** | **82.25** |

**Table 2 Changes of pediatric beds in different levels of hospitals in 11 districts, Guangzhou**

| District | Year | Primary hospitals | |  | Secondary hospitals | |  | Tertiary hospitals | |  | All | |
| --- | --- | --- | --- | --- | --- | --- | --- | --- | --- | --- | --- | --- |
|  |  | Pediatric beds | Growth rate (%) |  | Pediatric beds | Growth rate (%) |  | Pediatric beds | Growth rate (%) |  | Pediatric beds | Growth rate (%) |
| Liwan | 2010 | 2 |  |  | 36 |  |  | 19 |  |  | 57 |  |
|  | 2014 | 2 | 0.00 |  | 42 | 16.67 |  | 89 | 368.42 |  | 133 | 133.33 |
|  | 2019 | 5 | 150.00 |  | 109 | 202.78 |  | 123 | 547.37 |  | 237 | 315.79 |
| Yuexiu | 2010 | 0 |  |  | 71 |  |  | 533 |  |  | 604 |  |
|  | 2014 | 0 |  |  | 113 | 59.15 |  | 563 | 5.63 |  | 676 | 11.92 |
|  | 2019 | 0 |  |  | 113 | 59.15 |  | 630 | 18.20 |  | 743 | 23.01 |
| Haizhu | 2010 | 0 |  |  | 41 |  |  | 446 |  |  | 487 |  |
|  | 2014 | 0 |  |  | 57 | 39.02 |  | 354 | -20.63 |  | 411 | -15.61 |
|  | 2019 | 20 |  |  | 42 | 2.44 |  | 606 | 35.87 |  | 668 | 37.17 |
| Tianhe | 2010 | 13 |  |  | 42 |  |  | 882 |  |  | 937 |  |
|  | 2014 | 7 | -46.15 |  | 52 | 23.81 |  | 713 | -19.16 |  | 772 | -17.61 |
|  | 2019 | 11 | -15.38 |  | 66 | 57.14 |  | 1000 | 13.38 |  | 1077 | 14.94 |
| Baiyun | 2010 | 23 |  |  | 111 |  |  | 113 |  |  | 247 |  |
|  | 2014 | 52 | 126.09 |  | 165 | 48.65 |  | 207 | 83.19 |  | 424 | 71.66 |
|  | 2019 | 98 | 326.09 |  | 208 | 87.39 |  | 365 | 223.01 |  | 671 | 171.66 |
| Huangpu | 2010 | 55 |  |  | 111 |  |  | 0 |  |  | 166 |  |
|  | 2014 | 10 | -81.82 |  | 68 | -38.74 |  | 84 |  |  | 162 | -2.41 |
|  | 2019 | 20 | -63.64 |  | 69 | -37.84 |  | 173 |  |  | 262 | 57.83 |
| Panyu | 2010 | 65 |  |  | 354 |  |  | 0 |  |  | 419 |  |
|  | 2014 | 81 | 24.62 |  | 32 | -90.96 |  | 613 |  |  | 726 | 73.27 |
|  | 2019 | 146 | 124.62 |  | 29 | -91.81 |  | 788 |  |  | 963 | 129.83 |
| Huadu | 2010 | 46 |  |  | 168 |  |  | 0 |  |  | 214 |  |
|  | 2014 | 61 | 32.61 |  | 175 | 4.17 |  | 102 |  |  | 338 | 57.94 |
|  | 2019 | 76 | 65.22 |  | 82 | -51.19 |  | 245 |  |  | 403 | 88.32 |
| Nansha | 2010 | 76 |  |  | 0 |  |  | 0 |  |  | 76 |  |
|  | 2014 | 46 | -39.47 |  | 62 |  |  | 20 |  |  | 128 | 68.42 |
|  | 2019 | 73 | -3.95 |  | 89 |  |  | 44 |  |  | 206 | 171.05 |
| Zengcheng | 2010 | 68 |  |  | 119 |  |  | 0 |  |  | 187 |  |
|  | 2014 | 72 | 5.88 |  | 94 | -21.01 |  | 45 |  |  | 211 | 12.83 |
|  | 2019 | 87 | 27.94 |  | 174 | 46.22 |  | 53 |  |  | 314 | 67.91 |
| Conghua | 2010 | 6 |  |  | 48 |  |  | 0 |  |  | 54 |  |
|  | 2014 | 0 | -100.00 |  | 100 | 108.33 |  | 0 |  |  | 100 | 85.19 |
|  | 2019 | 0 |  |  | 0 | -100.00 |  | 90 |  |  | 90 | 66.67 |
| **Total** | **2010** | **354** |  |  | **1101** |  | **0** | **1993** |  | **0** | **3448** |  |
|  | **2014** | **331** | **-6.50** |  | **960** | **-12.81** | **0** | **2790** | **39.99** | **0** | **4081** | **18.36** |
|  | **2019** | **536** | **51.41** |  | **981** | **-10.90** | **0** | **4117** | **106.57** | **0** | **5634** | **63.40** |

**Table 3 Sensitivity analysis by Fixed-effects Poisson regression models**

|  | *Parameter* | *Estimate* | *SE* | *IRR (95%CI)* | *p-value* |
| --- | --- | --- | --- | --- | --- |
| U5MR | Pediatrician ^a^ density^a^ | -0.28 | 0.06 | 0.75(0.67-0.85) | <0.01 |
|  | Pediatrician ^b^ density^b^ | -0.34 | 0.07 | 0.71(0.62-0.82) | <0.01 |
|  | Pediatrician ^c^ density^c^ | -0.43 | 0.09 | 0.65(0.55-0.78) | <0.01 |
| IMR | Pediatrician ^a^ density^a^ | -0.20 | 0.07 | 0.82(0.72-0.94) | <0.01 |
|  | Pediatrician ^b^ density^b^ | -0.24 | 0.08 | 0.79(0.68-0.92) | <0.01 |
|  | Pediatrician ^c^ density^c^ | -0.30 | 0.10 | 0.74(0.61-0.90) | <0.01 |

Fixed-effects Poisson regression models controlled for time-invariant district-specific factors were used to conduct the sensitivity analysis.

The superscript (a, b, c) that represent Pediatrician density were estimated by three ratios (4:6, 5:5, 6:4) of resident children to migrant children.

Abbreviation: U5MR, under-5 mortality rate; IMR, infant mortality rate; SE, standard error; IRR, incidence rate ratio; CI, confidence interval
